# Aflibercept therapy for exudative age-related macular degeneration resistant to bevacizumab and ranibizumab

**DOI:** 10.1186/s40942-021-00299-4

**Published:** 2021-04-01

**Authors:** Mohamed A. Hamid, Nizar S. Abdelfattah, Jamshid Salamzadeh, Sahar T. A. Abdelaziz, Ahmed M. Sabry, Khaled M. Mourad, Azza A. Shehab, Baruch D. Kuppermann

**Affiliations:** 1 Department of Ophthalmology, Gavin Herbert Eye Institute, University of California Irvine, Irvine, CA USA; 2 Department of Ophthalmology, Minia University, Minia, 61111 Egypt; 3 Department of Ophthalmology, David Geffen School of Medicine, University of California Los Angeles, Los Angeles, CA USA; 4 Department of Clinical Pharmacy, and Pharmacoeconomy and Pharma-Management, School of Pharmacy, Shahid Beheshti University of Medical Sciences, Tehran, Iran

**Keywords:** Age-related macular degeneration (AMD), Aflibercept, Anti-vascular endothelial growth factor (VEGF)

## Abstract

**Background:**

Despite the good outcomes achieved with intravitreal angiogenic therapy, a subset of neovascular age-related macular degeneration (AMD) patients experience resistance to therapy after repeated injections. Switching drugs could offer benefit to this group of patients.

**Purpose:**

To determine visual and anatomical outcomes in a cohort of neovascular AMD patients resistant to repeated injections of bevacizumab/ranibizumab after switching to aflibercept therapy.

**Methods:**

This was a retrospective chart review of patients who had a diagnosis of neovascular AMD and persistent intraretinal (IRF) and/or subretinal fluid (SRF) on optical coherence tomography (OCT) for at least 3 months despite monthly bevacizumab and/or ranibizumab injections prior to transition to aflibercept. We reviewed patients’ records and OCT images obtained at baseline, 1, 3, 6 and 12 months after transition to aflibercept. Data collected included demographics, best-corrected visual acuity (BCVA), number of injections received and the occurrence of any adverse events. Studied OCT parameters included central macular thickness (CMT) values and the presence or absence of SRF, IRF and/or pigment epithelial detachment (PED) at each visit.

**Results:**

We included 53 eyes of 48 patients. Mean change in BCVA from baseline was 0.05 ± 0.13 (P = 0.01) at M1, 0.04 ± 0.16 (P = 0.08) at M3, 0.01 ± 0.22 (P = 0.9) at M6, and 0.02 ± 0.28 (P = 1) at M12, while the mean change in CMT from baseline was 64 ± 75 μm (P < 0.0001) at M1, 42 ± 85 μm (P = 0.002) at M3, 47 ± 69 μm (P < 0.0001) at M6, and 46 ± 99 μm (P = 0.001) at M12. The percentage of eyes with SRF decreased from 77.4% at baseline to 39.6% at M1, then increased to 47.2% at M3, then decreased to 43.4% at M6, and to 41.5% at M12 (All p < 0.001, compared to baseline). Compared to baseline, there was a statistically significant decrease in the percentage of eyes having IRF from 47.2 to 20.8% at M1 (p < 0.001), 30.2% at M3, 24.5% at M6 and 26.4% at M12 (p < 0.01, each). The number of bevacizumab and/or ranibizumab injections (7.36 ± 1.85) was significantly higher than that of aflibercept (6.47 ± 2.45, p = 0.001). A significant direct relationship between CMT reduction and BCVA improvement was demonstrated at M1 (p = 0.01, r = 0.36), M3 (p = 0.03, r = 0.30) and M12 (p = 0.03, r = 0.30). Eyes with IRF had significantly poorer BCVA than eyes without IRF at baseline (p = 0.02) and M3 (p = 0.04).

**Conclusion:**

Switching to intravitreal aflibercept therapy in a cohort of neovascular AMD patients resistant to chronic bevacizumab and/or ranibizumab injections can lead to significant visual improvement in the short term and sustained reduction of central macular thickness over 1 year of followup.

## Background

Age-related macular degeneration (AMD) is the most common cause of irreversible vision loss in developed countries among individuals aged 50 years and older [1]. The neovascular form of late AMD affects only 10% of patients, but is responsible for most cases of severe vision loss due to the disease [2].

The introduction of vascular endothelial growth factor (VEGF) inhibitors has led many neovascular AMD patients to achieve meaningful gains in visual acuity or at least maintain a stable vision. However, some patients exhibit suboptimal or nonresponse to anti-VEGF injections, while others experience a slowly diminishing effect of treatment after chronic injections for extended periods [3]. The CATT study showed that 51.5–67.4% of patients still had fluid on optical coherence tomography (OCT) despite having received monthly ranibizumab or bevacizumab injections for 2 years [4].

Aflibercept (Eylea, Regeneron, Inc., Tarrytown, NY, USA) is a more recent addition to the anti-VEGF armamentarium. It has a higher affinity for VEGF-A than bevacizumab and ranibizumab. Unlike the 2 aforementioned drugs, it can also bind VEGF-B and placental growth factor (PlGF) [5]. The most recent addition to anti-VEGF drugs has been brolucizumab; an antibody fragment that can inhibit all isoforms of VEGF-A. It is the smallest of the anti-VEGF drugs which allows for the administration of a more concentrated volume of the drug per single dose [6]. Several studies have suggested that switching therapies to aflibercept might be beneficial for patients resistant to chronic injections of bevacizumab and/or ranibizumab [7–11].

The purpose of this study is to determine anatomical and visual outcomes in a cohort of neovascular AMD patients resistant to repeated bevacizumab and/or ranibizumab injections that were switched to aflibercept therapy.

## Methods

### Study design

We retrospectively reviewed the charts of neovascular AMD patients who had previously been treated with ranibizumab and/or bevacizumab and then converted to aflibercept between December 2011, and July 2016.

The study protocol was approved by the Institutional Review Board of the University of California Irvine. The study complied with the Health Insurance Portability and Accountability Act (HIPPA) of 1996 and conformed to the tenets of the Declaration of Helsinki.

### Study population

Patients who were 50 years or older were included if they had a diagnosis of neovascular AMD (NAMD) and persistent intraretinal (IRF) and/or subretinal fluid (SRF) on spectral domain optical coherence tomography (SD-OCT) for a minimum of 3 months despite monthly bevacizumab and/or ranibizumab injections prior to transition to aflibercept therapy. Patients must have had a minimum of 6 bevacizumab/ranibizumab injections in the year prior to transition and a minimum of 3 aflibercept injections in the year after. An interval of at least 28 days was allowed between the last bevacizumab/ranibizumab injection and the first aflibercept treatment. A minimum follow-up period of 12 months was required. Patients were excluded if they had a co-existing confounding retinal pathology (e.g. polypoidal choroidal vasculopathy, central serous chorioretinopathy, diabetic macular edema, retinal vein occlusion, significant vitreoretinal interface abnormalities, hereditary retinal dystrophies) or a confounding cause of vision loss (e.g. optic neuropathy, uncontrolled IOP > 25 mmHg, diabetic retinopathy more severe than mild nonproliferative changes, retinal detachment). Patients who had subfoveal geographic atrophy, significant disciform scarring (> 50% of the lesion), RPE tear, or subretinal hemorrhage (SRH) involving > 1-disc area (DA) of the fovea at baseline were also excluded. Patients with history of previous intraocular surgery, apart from uneventful cataract extraction and intraocular lens implantation, in the last 6 months before transition, any other treatment for AMD than bevacizumab/ranibizumab and nutritional supplements before baseline, or aflibercept and nutritional supplements during follow up were also excluded.

### Treatment and follow-up schedule

All patients underwent a comprehensive ophthalmologic examination at baseline and at each follow-up visit. SD-OCT imaging was performed at each clinic visit using a Spectralis (Heidelberg Engineering, Inc., Franklin, MA) system.

Patients were treated using a modified *pro re nata* (PRN) regimen that does not include initial monthly loading. Patients were scheduled for regular visits every 4–6 weeks. Anti-VEGF treatment was administered if best-corrected visual acuity (BCVA) decreased by ≥ 1 line on Snellen chart, there was evidence of IRF and/or SRF on SD-OCT or a new intraretinal/subretinal hemorrhage was detected on fundus examination.

### OCT-based parameters

OCT-based parameters were obtained from images taken at baseline and follow-up visits at 1, 3, 6 and 12 months.

Central macular thickness (CMT) values were obtained using the integrated software in Spectralis OCT machine. When segmentation errors precluded accurate automated measurement of CMT, it was measured manually using the caliper function from the foveal depression to Bruch’s membrane. Where PED obscured the outer retinal layers, CMT was measured to the estimated location of Bruch’s membrane.

Intraretinal fluid (IRF), subretinal fluid (SRF) and pigment epithelial detachment (PED) were categorized into “present” or “absent” based on review of entire volume scans.

### Outcome measures

Snellen visual acuities were converted to logarithm of the minimal angle of resolution (logMAR) for statistical analysis. We defined a BCVA difference ≥ 0.1 logMAR as a clinically important difference, while a difference < 0.1 logMAR represented a clinically stable BCVA [12]. At least a 10% change between CMT measurements was considered clinically significant.

The primary outcome measures of the study were the change from baseline in BCVA and CMT at 1, 3, 6 and 12 months of followup.

The secondary outcome measures were:

1. The proportion of eyes that were dry at each time point.
2. The proportion of eyes that had stable VA, gain of ≥ 1 line and loss of ≥ 1 line at each time point.
3. The injection frequency in the 12-month follow-up period compared to that in the 12-month period prior to conversion to aflibercept.
4. Correlation between OCT-based parameters (CMT, presence or absence of PED, SRF or IRF) and BCVA at each time point.
5. Analysis of baseline factors (age, gender, right vs left eye, BCVA, CMT, presence of PED, SRF, IRF, presence or absence of diabetes, and phakic status) that could predict improvement of BCVA and/or CMT at final follow up.
6. The incidence of ocular and systemic adverse events.

### Statistical analyses

A one-way repeated measures ANOVA was conducted to determine whether there was a statistically significant difference in BCVA or CMT over the course of a 12-month aflibercept switch intervention. Epsilon (ε) was used to correct the one-way repeated measures ANOVA. Cochran's Q test was run to determine if the percentage of eyes that had PED, SRF or IRF significantly changed during follow-up. Exact McNemar's tests were used to assess all pairwise comparisons. A Bonferroni correction was applied with statistical significance accepted at p < 0.167. The non-parametric Wilcoxon Signed Rank test was used to compare the differences in number of injections needed before and after the switch. A univariate analysis of variables was conducted to determine the effect of multiple risk factors on change in BCVA or CMT by the end of follow-up. Spearman’s rank-order correlation test was used to investigate any possible correlation between CMT and BCVA at each time point. The non-parametric “Mann–Whitney U” test was used to detect any correlation between the presence or absence of IRF or SRF and BCVA at each time point. All statistical analyses were done using IBM SPSS Statistics for Windows, Version 25.0 (IBM Corp., Armonk, NY, USA).

## Results

### Demographics

We included 53 eyes of 48 patients. Only one eye was included for 43 patients, while both eyes were included for 5 patients. The mean age for the study population was 81.8 ± 7.9 years (range: 64–96 years). Eighteen patients were males (37.5%) and 30 were females (62.5%). Table 1 summarizes baseline characteristics of the study cohort.

**Table 1:** Baseline characteristics of the study population

Variable		Value
Age^a^	Mean ± SD	81.8 ± 7.9 (64 to 96)
Sex^b^	Male	18 (37.5%)
	Female	30 (62.5%)
Eye^b^	OD	25 (47.2%)
	OS	28 (52.8%)
Laterality^b^	Unilateral	43 (89.6%)
	Bilateral	5 (10.4%)
DM^b^	Nondiabetic	40 (83.3%)
	Diabetic	8 (16.7%)
Phakic status^b^	Pseudophakic	37 (69.8%)
	Phakic	16 (30.2%)
Legacy^b^	L-Bevacizumab	10 (19.2%)
	L-Ranibizumab	42 (80.8%)

*OD* right eye, *OS* left eye, *DM* diabetes mellitus, *L-Bevacizumab* Legacy-Bevacizumab, *L-Ranibizumab* Legacy-Ranibizumab

^a^Mean ± SD

^b^Number (percentage)

### Visual outcomes

**Fig. 1:**
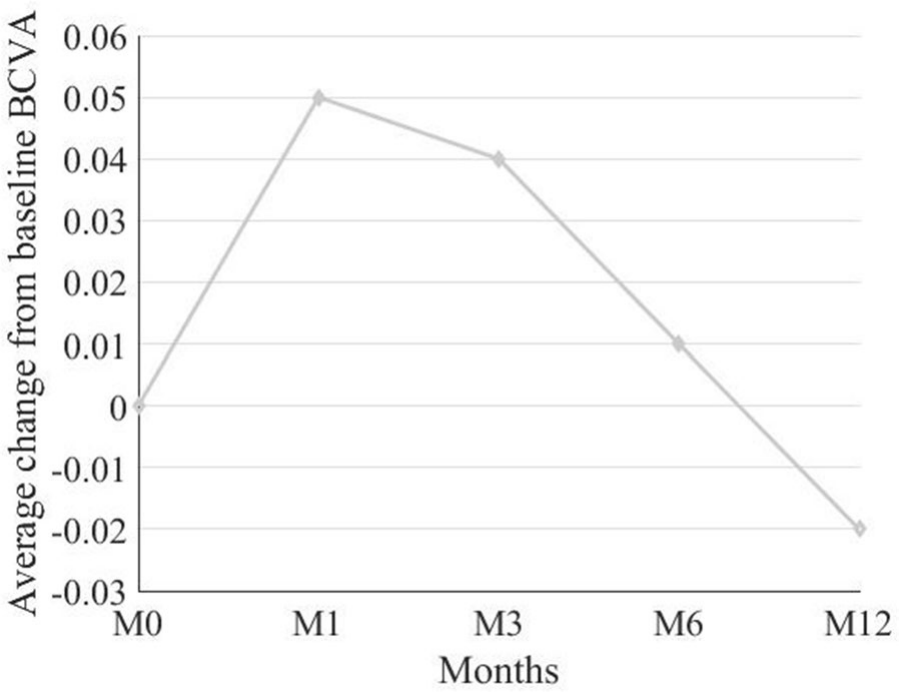
Average change from baseline best-corrected visual acuity (BCVA) at each time point; BCVA values are displayed in -Log MAR scale; M0: Baseline, M1: Month 1; M3: Month 3; M6: Month 6; M12: Month 12 of followup

The mean BCVA was 0.45 ± 0.28 at baseline, 0.4 ± 0.27 at M1, 0.41 ± 0.31 at M3, 0.44 ± 0.35 at M6, and 0.47 ± 0.39 at M12. Mean change in BCVA from baseline was 0.05 ± 0.13 (6%), 95% CI (0.09, 0.01), P = 0.01 at M1, 0.04 ± 0.16 (11%), 95% CI (0.08, 0.01), P = 0.08 at M3, 0.01 ± 0.22 (1%), 95% CI (0.07, 0.05), P = 0.9 at M6, and 0.02 ± 0.28 (7%), 95% CI (0.06, 1), P = 1 at M12 (Table 2; Fig. 1).

**Table 2:** Change in BCVA at each time point following transition to aflibercept therapy

Time point	BCVA^a^ (Mean ± SD)	Change from Baseline (Mean ± SD)	Change from Baseline (%)	P-value^b^	95% CI	
					Lower	Upper
Baseline	0.45 ± 0.28					
Month 1	0.4 ± 0.27	− 0.05 ± 0.13	6	0.01	− 0.09	-0.01
Month 3	0.41 ± 0.31	− 0.04 ± 0.16	11	0.079	− 0.08	0.01
Month 6	0.44 ± 0.35	− 0.01 ± 0.22		0.929	− 0.07	0.05
Month 12	0.47 ± 0.39	0.02 ± 0.28	7	1.00	0.06	0.1

*BCVA* best-corrected visual acuity, *CI* confidence interval

^a^logMAR, log_10_ of reciprocal of Snellen visual acuity

^b^ Based on hybrid linear mixed model, adjusted for multiple comparisons based on Sidak method

The proportion of eyes that gained ≥ 1 line was 21 (39.6%), 23 (43.4%), 23 (43.4%), and 20 (37.7%) at M1, 3, 6 and 12, respectively. A stable BCVA was maintained in 23 (43.4%), 16 (30.2%), 13 (24.5%), and 11 (20.8%) eyes at M1, 3, 6 and 12, respectively. BCVA loss ≥ 1 line was observed in 9 (17.0%), 14 (26.4%), 17 (32.1%), and 22 (41.5%) eyes at M1, 3, 6 and 12, respectively.

### Anatomical outcomes

The mean CMT was 387 ± 99 μm at baseline, 326 ± 90 μm at M1, 345 ± 88 μm at M3, 341 ± 96 μm at M6, and 342 ± 96 μm at M12.

**Fig. 2:**
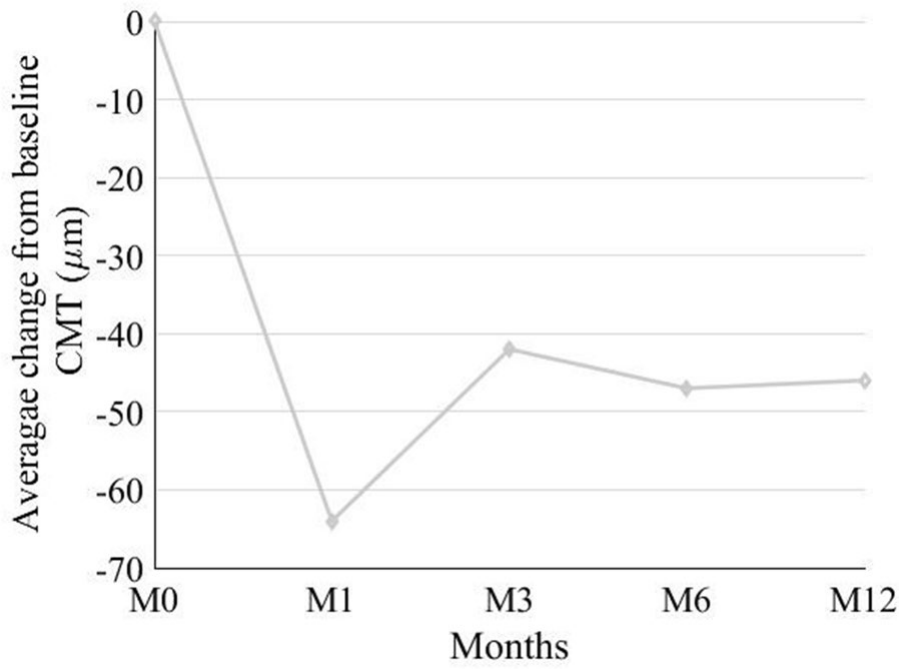
Average change from baseline central macular thickness (CMT) at each time point; CMT values are displayed in µm; M0: Baseline, M1: Month 1; M3: Month 3; M6: Month 6; M12: Month 12 of followup

**Fig. 3:**
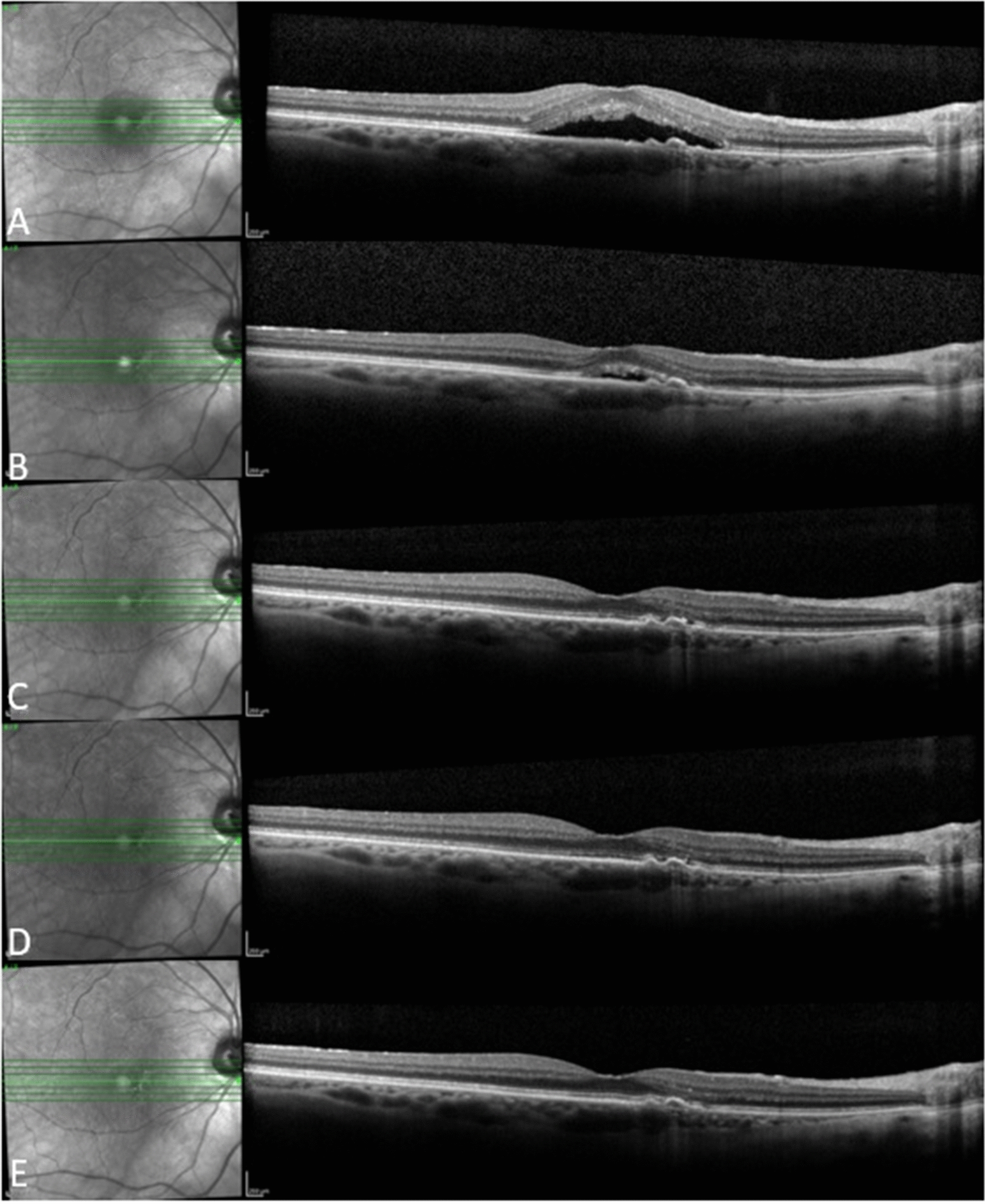
A 70-year-old female patient who received 6 ranibizumab injections in the year before baseline and 5 aflibercept injections in 1 year of follow up. She showed good response to aflibercept with complete resolution of subretinal fluid at Month 3 that was maintained through Month 12. **a** Baseline, **b** Month 1, **c** Month 3, **d** Month 6, **e** Month 12

Mean change in CMT from baseline was 64 ± 75 μm (15%), 95% CI (− 85, -43), P < 0.0001 at M1, 42 ± 85 μm (8%), 95% CI (− 66, − 18), P = 0.002 at M3, 47 ± 69 μm (11%), 95% CI (− 66, − 28), P < 0.0001 at M6, and 46 ± 99 μm (9%), 95% CI (− 73, − 18), P = 0.001 at M12 (Table 3; Figs. 2, 3).

**Table 3:** Change in CMT at each time point following transition to aflibercept therapy

Time point	CMT^a^ (Mean ± SD)	Change from Baseline (Mean ± SD)	Change from Baseline (%)	P-value^b^	95% CI	
					Lower	Upper
Baseline	387 ± 99					
Month 1	326 ± 90	− 64 ± 75	15	< 0.0001	85	− 43
Month 3	345 ± 88	− 42 ± 85	8	0.002	− 66	− 18
Month 6	341 ± 96	− 47 ± 69	11	< 0.0001	− 66	− 28
Month 12	342 ± 96	− 46 ± 99	9	0.001	− 73	− 18

*CMT* central macular thickness, CI: confidence interval

^a^ μM

^b^ Based on hybrid linear mixed model, adjusted for multiple comparisons based on Sidak method

The proportion of eyes that had ≥ 10% reduction in CMT was 29 (54.7%), 20 (37.7%), 25 (47.2%), and 25 eyes (47.2%) at M1, 3, 6 and 12, respectively. Stability in CMT was achieved in 24 (45.3%), 28 (52.8%), 24 (45.3%), and 19 eyes (35.8%) at M1, 3, 6 and 12, respectively. An increase in CMT of ≥ 10% was observed in 0, 5 (9.4%), 4 (7.5%), and 9 eyes (17.0%) at M1, 3, 6, and 12, respectively.

### Other OCT-based parameters

#### Pigment epithelial detachment (PED)

At baseline, all study eyes had a PED on OCT. Only one eye had resolved PED at M12 out of the entire cohort, causing a non-statistically significant difference at the different time points.

#### Subretinal fluid (SRF)

At baseline, 41 eyes (77.4%) of the examined cohort had SRF on SD-OCT. This percentage decreased to 39.6% (21 eyes) at M1, then increased to 47.2% (25 eyes) at M3, then decreased to 43.4% (23 eyes) at M6, and further to 41.5% (22 eyes) at M12. Compared to baseline, the percentage of eyes with SRF significantly decreased at all time points (p < 0.001).

#### Intraretinal fluid (IRF)

At baseline, 25 study eyes (47.2%) had IRF as detected on SD-OCT. The percentage of eyes having IRF decreased to 20.8% (11 eyes) at M1, then increased to 30.2% (16 eyes) at M3, then decreased to 24.5% (13 eyes) at M6 and increased to 26.4% (14 eyes) at M12. Compared to baseline, there was a statistically significant decrease in the percentage of eyes having IRF at M1 (p < 0.001), M3, M6 and M12 (p < 0.01, each).

### Frequency of intravitreal injections

Patients received a mean of 7.36 ± 1.85 bevacizumab/ranibizumab injections (median 6, IR 6.0–8.5) in the year prior to transition, and a mean of 6.47 ± 2.45 aflibercept injections (median 6, IR 5.0–8.0) in the following year. Overall, the number of bevacizumab and/or ranibizumab injections was significantly higher than that of aflibercept (p = 0.001).

### Predictors of change in BCVA or CMT from baseline to month-12

We studied the effect of age, sex, diabetes, laterality, baseline BCVA, baseline CMT, presence of PED, SRF, IRF, lens status and number of aflibercept injections on change in BCVA and change in CMT from baseline to M12. None was fond to significantly predict change in BCVA at month-12 (p = 0.16). Only baseline CMT was found to be positively correlated with change in CMT at M12 (p < 0.001). The more the baseline CMT, the more change in CMT was observed by month-12.

### Correlation between OCT parameters and BCVA at each time point

A significant direct relationship between CMT and BCVA was demonstrated at M1 (p = 0.01, r = 0.36), M3 (p = 0.03, r = 0.30) and M12 (p = 0.03, r = 0.30). As CMT decreased, BCVA improved and vice versa. No significant correlation was found at baseline (p = 0.12, r = 0.21) or M6 (p = 0.14, r = 0.21). A significant direct relationship between the presence of IRF and BCVA was also demonstrated only at baseline (p = 0.02) and M3 (p = 0.04). Eyes with IRF had poorer BCVA than eyes without IRF. No significant correlation was found at M1 (p = 0.21), M6 (p = 0.28) or M12 (p = 0.36).

Only one patient did not have PED at M12. Accordingly, comparison of BCVA between eyes with and without PED was not possible. No significant correlation was found between the presence of SRF and BCVA at any investigated time point.

### Adverse events

No ocular or systemic adverse events were found in our study population throughout one year of followup.

**Fig. 4:**
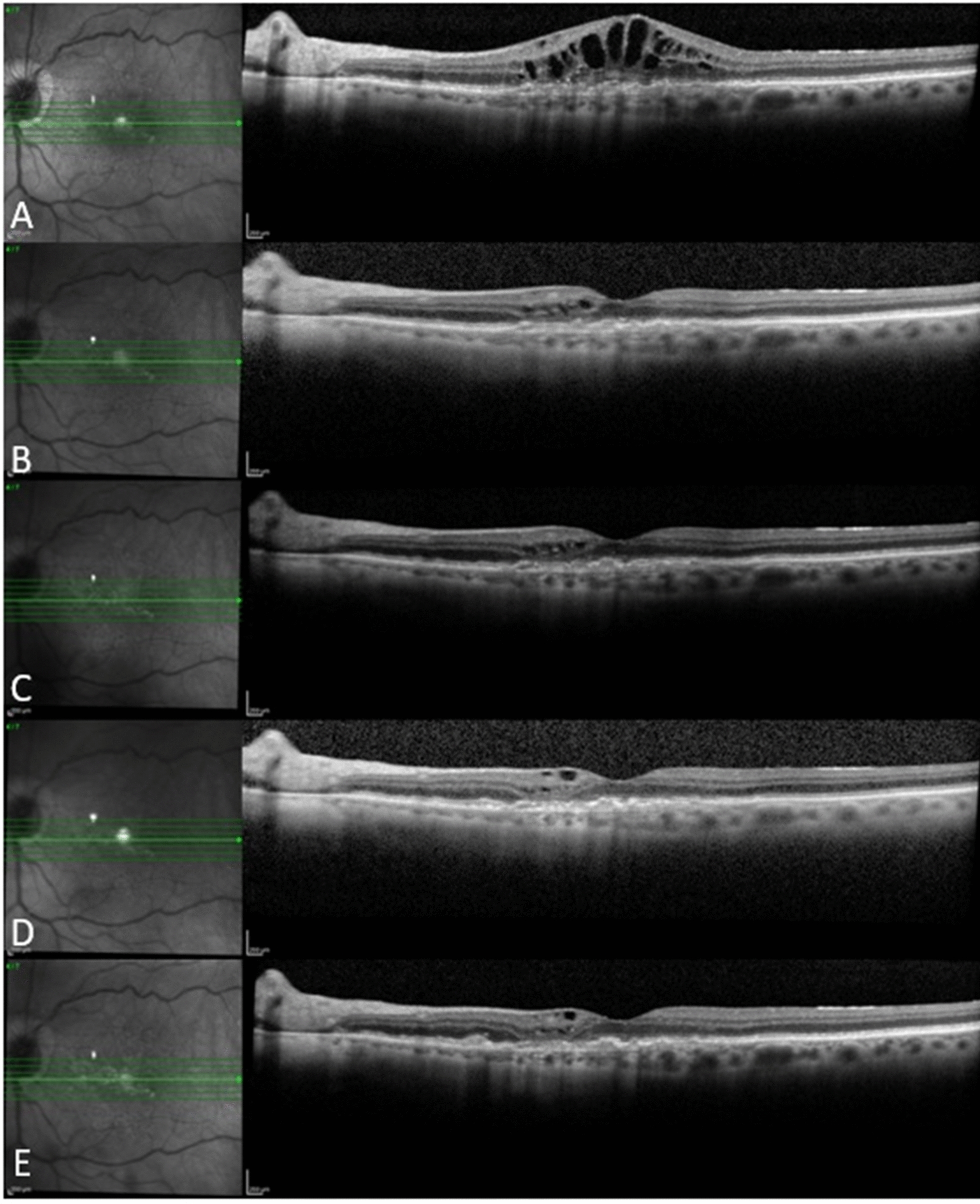
An 89-year-old female patient who received 5 bevacizumab and 6 ranibizumab injections in the year before baseline and 8 aflibercept injections in 1 year of follow up. She showed good response to aflibercept with marked improvement of intraretinal fluid after the first aflibercept injection followed by gradual resolution thereafter, with residual few extrafoveal cysts at last follow-up visit. **a** Baseline, **b** Month 1, **c** Month 3, **d** Month 6, **e** Month 12

**Fig. 5:**
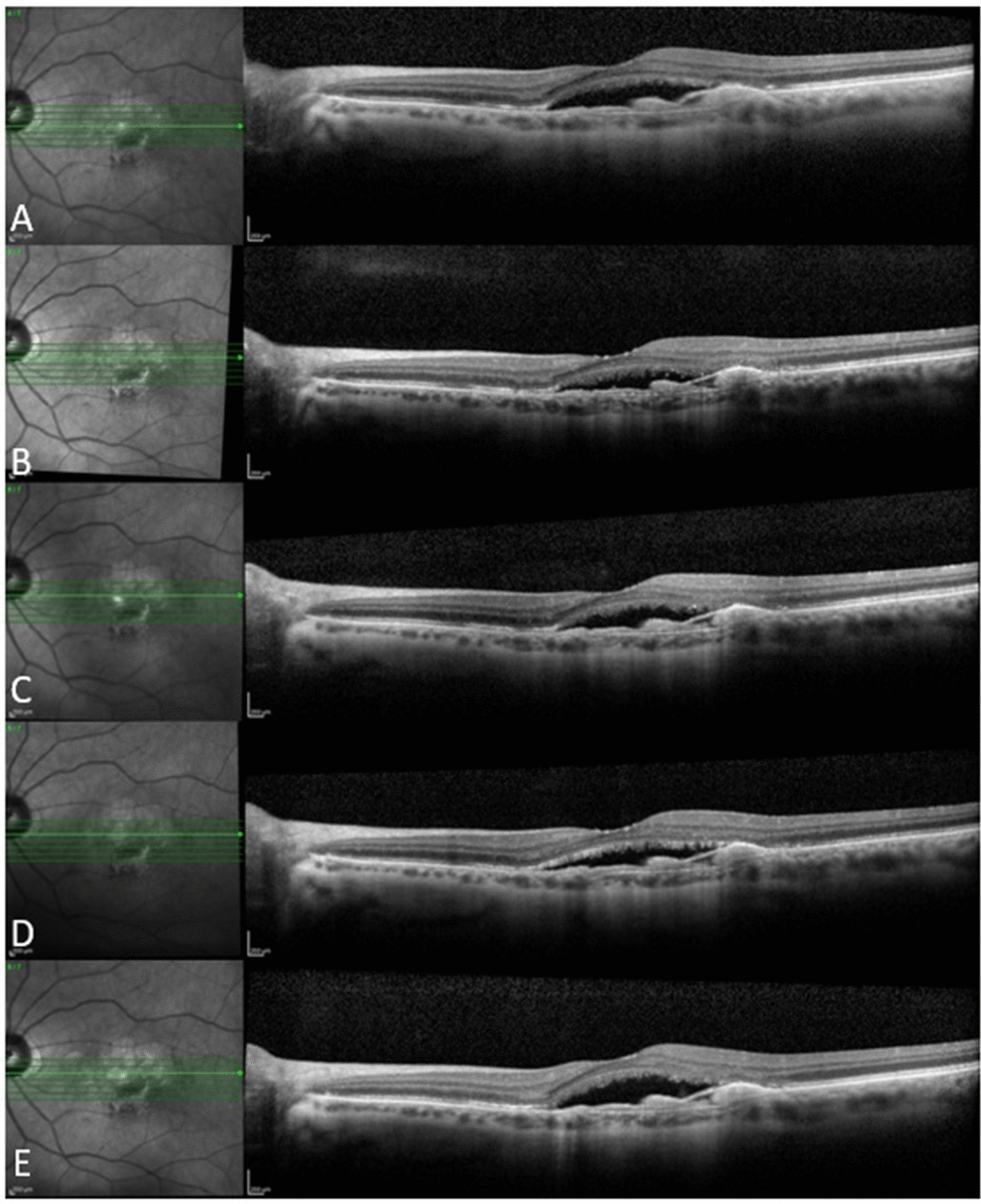
A 72-year-old female patient who received 12 ranibizumab injections in the year before baseline and 10 aflibercept injections in 1 year of follow up. The patient had poor response to aflibercept with persistence of subretinal fluid noted at all follow-up visits. **a** Baseline, **b** Month 1, **c** Month 3, **d** Month 6, **e **Month 12

Figures 3, 4 and 5 show 3 case examples from our study cohort.

## Discussion

Despite the dramatic breakthrough that anti-VEGF agents have shown in NAMD treatment, some patients have poor or nonresponse to anti-VEGF therapy or experience a slow loss of efficacy of anti-VEGF agents after repeated administration over time [4].

Previously, many researchers considered patients with persistent IRF or SRF after 3 initial injections to suffer from resistant AMD, which is mainly based on marked vision improvement after 3 monthly injections [13–17]. However, as the responses of > 30% of patients were delayed after 4 months of treatment in the MARINA and ANCHOR trials [18, 19], some investigators redefined refractory AMD as persistent exudation after at least 6 months of monthly treatment [3, 20, 21]. Generally, patients who show poor or no response to initial therapy, or had a successful initial response but experienced a slow loss of that response, are considered resistant to anti-angiogenic therapy [3, 22].

Our cohort of patients had a mean age of 81.8 years and 62.5% were females, concordant with previous studies [23]. Owing to the retrospective design of our study, we tried to ensure the validity of our results by applying stringent inclusion and exclusion criteria. A minimum period of 28 days was allowed between after the last bevacizumab/ranibizumab injection before transition, so that the drugs would be washed out and not compete with aflibercept for the same target [5]. A minimum of 3 monthly injections before transition, and a total of 6 in the prior year, as well as a maximum of 42 day-interval between injections were required to make sure that our cohort was truly resistant to therapy and not being undertreated.

Our study patients were treated according to a modified PRN regimen which reflects the practice pattern in our institution during the study period. Although the use of a PRN dosing regimen was validated in major clinical trials such as CATT [24], the later introduction of a Treat-and-Extend (TAE) regimen provided a more efficient approach that requires less patient visits. The TREX-AMD trial demonstrated better visual outcomes in eyes that were maintained on a TAE regimen compared to those that were transitioned from a monthly or a TAE regimen to a PRN dosing regimen during the third year of the study [25]. A PRN regimen is a standard practice in our institution but might not be feasible in other practices that deal with patients who have different insurance coverage. Even though it is associated with less frequent injections compared to both fixed dosing and TAE regimens, it does not decrease the frequency of scheduled visits with healthcare providers. The monthly visits have their own cost and time burdens regardless of whether injections are administered during these visits [26]. The choice of TAE could have impacted the outcome of our study as this regimen achieves better control over fluid and could theoritically decrease the percentage of eyes that need to switch therapies.

A recent meta-analysis of 28 studies that enrolled a total number of 2,245 eyes with NAMD transitioned to aflibercept after being resistant to bevacizumab/ranibizumab has found considerable anatomical improvement, although visual improvement was limited [23]. They attributed the limited visual improvement to better baseline visual acuity and the chronic nature of disease in the studied patient population. Interestingly, even though the pooled analysis showed no significant visual improvement, prospective studies showed better visual outcomes that reached statistical significance compared to retrospective studies. This difference was attributed to the more structured protocols of prospective studies that reduced biases inherent to retrospective analyses. Other meta-analyses demonstrated the same effect of study design on visual outcomes [27]. Indeed, our retrospective review found the change in visual acuity from baseline to be only statistically significant at M1 and nonsignificant thereafter. The relatively good visual acuity at baseline might have resulted in a ceiling effect on visual improvement as well. Although visual acuity did not significantly improve after M1 compared to baseline, a meaningful gain of ≥ 1 line was achieved, and vision stability was maintained, in a significant proportion of eyes throughout the study period, which could have a significant impact on quality of life [28].

We demonstrated a significant reduction in CMT all follow-up visits. Even though CMT is the easiest way to quantify retinal changes, it is far from ideal. Among its drawbacks are being prone to segmentation errors and poor patient fixation, as well as lack of reproducibility across different OCT platforms. Its main limitation is its inability to capture small changes at the level of different pathologic constituents [29].

The percentage of eyes with SRF in our cohort significantly decreased at all timepoints compared to baseline. Although the detection of SRF on OCT is a criterion for retreatment in almost every major AMD trial, there has been a recent controversy as to whether some residual SRF could be tolerated [30]. It has been hypothesized that SRF leakage from the CNV complex, to some extent, might represent a compensatory mechanism to capitulate for the diminished choriocapillaris perfusion of the outer retina in advanced AMD [31]. The presence of residual SRF was associated with less incidence of macular atrophy and better visual outcomes in an analysis of the 2-year results of CATT [32]. In alignment with the previous findings, the FLUID study recently demonstrated that a relaxed Treat-and-Extend regimen that tolerated < 200 µm of SRF at the foveal center achieved similar visual gains with less frequent injections compared to an intensive regimen that tolerated no SRF [33].

The percentage of eyes with IRF in our study also significantly decreased at all follow-up visits compared to baseline. Eyes with IRF had poorer baseline vision compared to those without IRF. In the context of neovascular AMD, the presence of IRF usually harbors a poorer functional prognosis compared to SRF. Damage to the outer retinal blood barrier and the external limiting membrane tight junctions are considered responsible for the appearance of IRF [30]. Furthermore, the evaluation of IRF on OCT to guide retreatment decisions can be more complex. Other lesions that could mimic IRF resulting from active CNV exudation include degenerative cysts and outer retinal tubulations among many. These mimicking lesions are typically not responsive to anti-VEGF treatment and indicate irreversible damage to the neurosensory retina [29].

PED occurs in up to 62% of neovascular AMD cases [34]. It has been shown to respond poorly to all treatments for CNV including anti-VEGF therapy [35]. The reason is likely the deeper location of the RPE layer and its barrier effect against penetration of the drug into the subretinal space. This finding of relative difficulty in reducing the PED compared to SRF and IRF has been described in the literature [36–38]. Aflibercept has been reported to significantly reduce PED height and volume by 12–33% in previous studies, even in the absence of complete PED resolution [34]. It has also been demonstrated to effectively reduce the dimensions of PEDs resistant to other anti-VEGF agents [34, 35, 39–42]. In our analysis, only 1 eye had a resolved PED at M12. However, we did not quantify PED dimensions at any time point in our study.

We utilized a dichotomous approach when evaluating SRF, IRF and PED on OCT; either present or absent. Although we relied on this simplified qualitative approach to reflect real-world everyday clinical practice, we acknowledge that it is imperfect and prone to error and misinterpretation [43]. Recent studies have used sophisticated quantification methods of these OCT parameters including customized software and artificial intelligence-based algorithms [43–46]. An analysis of the FLUID study that quantified IRF and SRF using a deep learning algorithm found that IRF in the central 1 mm of the macula and SRF in the 1 to 6-mm ring were associated with BCVA reduction, however, SRF in the central mm and IRF in the 1 to 6-mm ring were not associated with such reduction. This demonstrates that the location of fluid, and not only its volume, is important in determining disease activity [43]. Adoption of such quantitative methods could yield more accurate results in future clinical trial as well as guide more informed treatment decisions in clinical practice.

The pivotal phase 3 VIEW 1 and 2 trials showed the noninferiority of bimonthly and monthly aflibercept to monthly ranibizumab [47]. However, there is lack of published data on comparing injection of frequency between aflibercept and bevacizumab/ranibizumab in a real-world setting with as-needed dosing regimens. Our study showed a significant, yet modest, median reduction of 1 injection after 1 year of transitioning to aflibercept. This comes in agreement with Chan et al.who demonstrated a reduction in the mean number of injections administered within a 6-month period from 6.5 to 5.4 in a cohort of resistant eyes that were switched from bevacizumab/ranibizumab to aflibercept [40]. Longer follow-up periods are required to determine if the frequency of required injections will continue to decline and, thus, have a meaningful impact on cost burdens.

The ASSESS study is the largest prospective trial to date that transitioned neovascular AMD patients from ranibizumab/bevacizumab to aflibercept using a fixed dosing regimen characterized by 3 monthly loading doses followed by a bimonthly regimen thereafter. They demonstrated significant visual and anatomical improvement at both 1 and 2 years. During the 3^rd^ year, the regimen was changed to a flexible PRN regimen that relied on physicians’ discretion. This resulted in gradual decline in vision and anatomical gains obtained during the first 2 years. These findings highlight the effects variable study designs could have on the final outcomes among other variables [10, 48].

In this study, CMT reduction and the absence of IRF significantly correlated with better visual outcomes at some, but not all time points. Such an inverse relationship between OCT results and vision outcome is logical. However, the lack of significant correlation between SRF presence and visual acuity, as well as between the other OCT-based parameters and vision at some time points, could be explained by the moderate level of mean visual acuity at baseline (mean 20/50) which likely resulted in a ceiling effect. Our findings are consistent with the reports of variable correlation coefficients between OCT-measured CMT and vision outcomes in previous studies.[49–52] Although OCT-based parameters are important for monitoring clinical progress with treatment, they cannot serve as surrogates for visual acuity measurements.

No ocular or systemic adverse events were encountered in any of the study eyes during 1 year of follow up. The meta-analysis by Spooner and colleagues reported similar findings in a total of 2,245 eyes. Most of the included studies reported no ocular or systemic adverse events [23].

The strengths of our study include a cohort of patients that was closely and uniformly monitored with ocular examination and high-quality SD-OCT, in addition to rigorous statistical analyses that included sample size consideration and Bonferroni adjustment for multiple testing. In addition, reliable and detailed records were available for all patients on our electronic medical record (EMR) database. A single investigator evaluated all OCT-based parameters in precisely the same manner.

The data of our study, however, need to be interpreted with caution, given the inherent limitations associated with a retrospective study. Other limitations include the non-standardized measurements of visual acuity, qualitative evaluation of OCT-based parameters and the lack of data beyond 12 months. Even though AMD is a common disease, our sample size is relatively small owing to our strict eligibility criteria and the period of our analysis which was limited by both the date of introduction of aflibercept into our practice and the implementation date of our EMR system that we used for the purpose of our study. Finally, in the absence of a comparison group; one that has met the criteria for switching but continued their original treatment, it is difficult to assert that any beneficial effects observed after switching in our study are in fact due to the new drug. Indeed, investigators from the CATT and DRCR.net have demonstrated significant functional and anatomical improvement in a cohort of patients who continued their originally assigned treatment despite meeting the criteria for switching to another anti-VEGF drug 3 or 6 months after starting therapy. We echo their recommendation that randomized controlled trials be conducted in the future to provide more robust conclusions on the effects of switching [53].

## Conclusions

In conclusion, we demonstrated that switching to intravitreal aflibercept therapy in a cohort of neovascular AMD patients resistant to chronic bevacizumab and/or ranibizumab injections can lead to significant visual improvement in the short term and sustained reduction of central macular thickness over 1 year of follow-up. Patients required less frequent intravitreal injections to maintain the visual and anatomical benefits.

## Data Availability

The datasets used and/or analyzed during the current study are available from the corresponding author on reasonable request.

## Abbreviations

AMD: Age-related macular degeneration
BCVA: Best-corrected visual acuity
CMT: Central macular thickness
CNV: Choroidal neovascularization
DA: Disc area
EMR: Electronic medical record
IOP: Intraocular pressure
IRF: Intraretinal fluid
LogMAR: Logarithm of minimum angle resolvable
NAMD: Neovascular age-related macular degeneration
OCT: Optical coherence tomography
PED: Pigment epithelial detachment
PlGF: Placental growth factor
PPV: Pars plana vitrectomy
PRN: *Pro re nata*
RPE: Retinal pigment epithelium
SD OCT: Spectral domain optical coherence tomography
SRF: Subretinal fluid
SRH: Subretinal hemorrhage
TAE: Treat and extend
VEGF: Vascular endothelial growth factor
